# The CUSUM chart method as a tool for continuous monitoring of clinical outcomes using routinely collected data

**DOI:** 10.1186/1471-2288-7-46

**Published:** 2007-11-03

**Authors:** Thabani Sibanda, Nokuthaba Sibanda

**Affiliations:** 1 Epsom & St Helier Univesity Hospitals NHS Trust, St Helier Hospital, Wrythe Lane, Carshalton, Surrey, SM5 1AA, UK; 2 London School of Hygiene & Tropical Medicine, London WC2A 3PE, UK

## Abstract

**Background:**

The lack of robust systems for monitoring quality in healthcare has been highlighted. Statistical process control (SPC) methods, utilizing the increasingly available routinely collected electronic patient records, could be used in creating surveillance systems that could lead to rapid detection of periods of deteriorating standards. We aimed to develop and test a CUmulative SUM (CUSUM) based surveillance system that could be used in continuous monitoring of clinical outcomes, using routinely collected data. The low Apgar score (5 minute Apgar score < 7) was used as an example outcome.

**Method:**

A surveillance system based on the Observed minus Expected (O-E) as well as the 2-sided Log-Likelihood CUSUM charts was developed. The Log-Likelihood chart was designed to detect a 50% rise (deterioration) and halving (improvement) in the odds of low Apgar scores. Baseline rates were calculated from data for 2001 to 2004, and were used to monitor deliveries for 2005. Deliveries for nulliparous and multiparous women were monitored separately. All analyses were retrospective.

**Results:**

The CUSUM system detected periods of increased rates of low Apgar scores for each of the nulliparous and multiparous cohorts. The overall rate for 2005 was eventually found to be 0.67%, which was higher than the baseline reference rate of 0.44% from 2001 to 2004.

**Conclusion:**

CUSUM methods can be used in continuous monitoring of clinical outcomes using routinely collected data. Used prospectively, they could lead to the prompt detection of periods of suboptimal standards.

## Background

Calls for robust systems for monitoring healthcare outcomes have been made [1]. The increasing use of electronic patient records in healthcare presents an opportunity for the development and application of real-time monitoring systems that can lead to the rapid detection of adverse trends in healthcare. Statistical process control (SPC) methods, developed and long used in quality control systems in the manufacturing industry [2], could become central to such efforts. We describe the design and retrospective application of a surveillance system in the continuous monitoring of clinical outcomes using an SPC tool known as the CUmulative SUM (CUSUM) chart, using routinely collected data. We used a neonatal clinical outcome, the low Apgar score (5 minute Apgar score < 7), as an example outcome.

### The CUmulative SUM (CUSUM) chart

The CUSUM chart method, first described by Page in 1954[3], is based on sequential monitoring of a cumulative performance measure over time. With several developments and adaptations, it has emerged as a suitable method for monitoring healthcare outcomes [4-8]. We selected and used the "Observed *minus *Expected" (O-E) and the Log-likelihood CUSUM chart methods in designing our surveillance tool.

#### Observed – Expected (O-E) CUSUM chart

This form of the CUSUM chart is a graphical representation of a running total of the difference between the number of observed adverse events and that expected according to a specified baseline reference rate. It is mathematically defined as:

*C*_*t *_= *C*_*t*-1 _+ (*X*_*t *_- *X*_0_),

where *X*_*t *_is the outcome measurement for subject *t *(Observed), and *X*_0 _is the baseline reference rate (Expected), hence O – E. In binary outcome measures, *X*_*t *_equals 0 for a successful or desired outcome, and 1 for an adverse outcome. The chart is a plot of the cumulative sum, *C*_*t*_, against *t*. Desired outcomes result in downward steps, and upward steps are produced when adverse outcomes are encountered. When the outcome rate is consistent with the baseline reference rate, the plot runs randomly about the horizontal line at zero. Although in its simple form, the O – E chart has no limits and therefore does not give a signal if and when the rate changes have become statistically significant, it serves to illustrate an overall general trend in the rates of the adverse outcome monitored.

#### The Log Likelihood CUSUM chart

The Log Likelihood CUSUM chart is a probability testing procedure that sequentially assesses whether the observed adverse outcome rate is consistent with a specified baseline reference rate. Each subject is given a weight *W*_*t*_, which is obtained as follows [7];

Wt={log⁡[ORA(1−pt+ORApt)]for an adverse outcomelog⁡[1(1−pt+ORApt)]for a successful or desired outcome,
 MathType@MTEF@5@5@+=feaafiart1ev1aaatCvAUfKttLearuWrP9MDH5MBPbIqV92AaeXatLxBI9gBaebbnrfifHhDYfgasaacPC6xNi=xI8qiVKYPFjYdHaVhbbf9v8qqaqFr0xc9vqFj0dXdbba91qpepeI8k8fiI+fsY=rqGqVepae9pg0db9vqaiVgFr0xfr=xfr=xc9adbaqaaeGacaGaaiaabeqaaeqabiWaaaGcbaGaem4vaC1aaSbaaSqaaiabdsha0bqabaGccqGH9aqpdaGabeqaauaabaqaceaaaeaacyGGSbaBcqGGVbWBcqGGNbWzdaWadaqaaKqbaoaalaaabaGaem4ta8KaemOuai1aaSbaaeaacqWGbbqqaeqaaaqaamaabmaabaGaeGymaeJaeyOeI0IaemiCaa3aaSbaaeaacqWG0baDaeqaaiabgUcaRiabd+eapjabdkfasnaaBaaabaGaemyqaeeabeaacqWGWbaCdaWgaaqaaiabdsha0bqabaaacaGLOaGaayzkaaaaaaGccaGLBbGaayzxaaGaeeOzayMaee4Ba8MaeeOCaiNaeeiiaaIaeeyyaeMaeeOBa4MaeeiiaaIaeeyyaeMaeeizaqMaeeODayNaeeyzauMaeeOCaiNaee4CamNaeeyzauMaeeiiaaIaee4Ba8MaeeyDauNaeeiDaqNaee4yamMaee4Ba8MaeeyBa0MaeeyzaugabaGagiiBaWMaei4Ba8Maei4zaC2aamWaaeaajuaGdaWcaaqaaiabigdaXaqaamaabmaabaGaeGymaeJaeyOeI0IaemiCaa3aaSbaaeaacqWG0baDaeqaaiabgUcaRiabd+eapjabdkfasnaaBaaabaGaemyqaeeabeaacqWGWbaCdaWgaaqaaiabdsha0bqabaaacaGLOaGaayzkaaaaaaGccaGLBbGaayzxaaGaeeOzayMaee4Ba8MaeeOCaiNaeeiiaaIaeeyyaeMaeeiiaaIaee4CamNaeeyDauNaee4yamMaee4yamMaeeyzauMaee4CamNaee4CamNaeeOzayMaeeyDauNaeeiBaWMaeeiiaaIaee4Ba8MaeeOCaiNaeeiiaaIaeeizaqMaeeyzauMaee4CamNaeeyAaKMaeeOCaiNaeeyzauMaeeizaqMaeeiiaaIaee4Ba8MaeeyDauNaeeiDaqNaee4yamMaee4Ba8MaeeyBa0MaeeyzaugaaiabcYcaSaGaay5Eaaaaaa@A8B9@

where *P*_*t *_is the baseline reference rate for subject *t*. *OR*_*A *_is a pre-specified odds ratio under the alternative hypotheses.

If desired, a two-sided CUSUM chart can be designed, where the upward chart is designed to detect an increase in the adverse outcome rate (*OR*_*A *_> 1), while the downward chart is designed to detect a reduction in the adverse outcome rate (*OR*_*A *_< 1). In the upward chart, Yt+
 MathType@MTEF@5@5@+=feaafiart1ev1aqatCvAUfKttLearuWrP9MDH5MBPbIqV92AaeXatLxBI9gBaebbnrfifHhDYfgasaacPC6xNi=xH8viVGI8Gi=hEeeu0xXdbba9frFj0xb9qqpG0dXdb9aspeI8k8fiI+fsY=rqGqVepae9pg0db9vqaiVgFr0xfr=xfr=xc9adbaqaaeGacaGaaiaabeqaaeqabiWaaaGcbaGaemywaK1aa0baaSqaaiabdsha0bqaaiabgUcaRaaaaaa@2F8F@ is plotted against *t*, where Yt+=max⁡(0,Yt++Wt+)
 MathType@MTEF@5@5@+=feaafiart1ev1aaatCvAUfKttLearuWrP9MDH5MBPbIqV92AaeXatLxBI9gBaebbnrfifHhDYfgasaacPC6xNi=xH8viVGI8Gi=hEeeu0xXdbba9frFj0xb9qqpG0dXdb9aspeI8k8fiI+fsY=rqGqVepae9pg0db9vqaiVgFr0xfr=xfr=xc9adbaqaaeGacaGaaiaabeqaaeqabiWaaaGcbaGaemywaK1aa0baaSqaaiabdsha0bqaaiabgUcaRaaakiabg2da9iGbc2gaTjabcggaHjabcIha4jabcIcaOiabicdaWiabcYcaSiabdMfaznaaDaaaleaacqWG0baDaeaacqGHRaWkaaGccqGHRaWkcqWGxbWvdaqhaaWcbaGaemiDaqhabaGaey4kaScaaOGaeiykaKcaaa@40AC@. The downward CUSUM plots Yt−
 MathType@MTEF@5@5@+=feaafiart1ev1aaatCvAUfKttLearuWrP9MDH5MBPbIqV92AaeXatLxBI9gBaebbnrfifHhDYfgasaacPC6xNi=xH8viVGI8Gi=hEeeu0xXdbba9frFj0xb9qqpG0dXdb9aspeI8k8fiI+fsY=rqGqVepae9pg0db9vqaiVgFr0xfr=xfr=xc9adbaqaaeGacaGaaiaabeqaaeqabiWaaaGcbaGaemywaK1aa0baaSqaaiabdsha0bqaaiabgkHiTaaaaaa@2F99@ against *t*, where Yt−=min⁡(0,Yt−−Wt−)
 MathType@MTEF@5@5@+=feaafiart1ev1aaatCvAUfKttLearuWrP9MDH5MBPbIqV92AaeXatLxBI9gBaebbnrfifHhDYfgasaacPC6xNi=xH8viVGI8Gi=hEeeu0xXdbba9frFj0xb9qqpG0dXdb9aspeI8k8fiI+fsY=rqGqVepae9pg0db9vqaiVgFr0xfr=xfr=xc9adbaqaaeGacaGaaiaabeqaaeqabiWaaaGcbaGaemywaK1aa0baaSqaaiabdsha0bqaaiabgkHiTaaakiabg2da9iGbc2gaTjabcMgaPjabc6gaUjabcIcaOiabicdaWiabcYcaSiabdMfaznaaDaaaleaacqWG0baDaeaacqGHsislaaGccqGHsislcqWGxbWvdaqhaaWcbaGaemiDaqhabaGaeyOeI0caaOGaeiykaKcaaa@40D4@. These CUSUM charts are thus restricted to always stay above or below zero, respectively.

Limits are determined and placed, with the CUSUM chart considered to signal when such a limit is crossed. The "signal" is an indication of sufficient evidence that the adverse outcome rate is no longer consistent with the baseline level. When this happens, the monitoring process is stopped to allow for appropriate previously agreed action to be taken. This response begins with checking and confirming the accuracy of the data before further investigations and subsequent changes are introduced. After this, monitoring is continued, either with the settings unchanged or with changes made as appropriate.

Limits have an inter dependence with the Average Run Length (ARL). The out-of control average run length (OC-ARL) is the average number of subjects required before the CUSUM chart signals when the level of performance is unacceptable, and the in-control average run length (IC-ARL) is the average number of consecutive subjects required for the CUSUM chart to signal despite the true rate being at an acceptable level.

### The clinical outcome: The low Apgar score

We selected a typical neonatal clinical outcome, the low Apgar score (5 minute Apgar score < 7), as an example outcome. The Apgar score is a convenient shorthand for reporting the status of newborn babies as well as the effect of resuscitation[9]. It is a zero to ten (0 – 10) aggregate score based on 5 parameters assessed in nearly all babies born in UK hospitals as well as the rest of the world. The low Apgar score (5 minute Apgar score < 7), has been identified as a "key outcome" to be used in assessing the role and impact of Electronic Fetal Monitoring (EFM) guideline [10], produced by the National Institute for Clinical Excellence (NICE). The Royal College of Obstetricians and Gynaecologists (RCOG) have also included it in a "trigger list" of outcomes to be monitored using the adverse incident reporting system [11]. It is also one of the components of the Adverse Outcome Index (AOI), a ten outcome system recently proposed for use in the assessment of quality of care in delivery units in the US [12].

Southmead Hospital, a District General Hospital (DGH) in Bristol with around 5000 deliveries a year, reported a 50% reduction in the rate of low Apgar scores in a subset of term (>37 weeks gestation) deliveries, following the introduction of regular training of all labour ward staff in the management of obstetric emergencies[13]. Their low Apgar score rate, which had been 0.86% in the 1998 to 1999 period, decreased to 0.44% after the introduction of the training programme in 2000. With new standards achieved, Southmead Hospital's data were ideal for testing a surveillance system that could be used in prospective monitoring of such clinical outcome in maternity units in the UK. Such a system would provide early warnings when rates of adverse outcomes are seen to be rising.

## Methods

Approval for the project was obtained from Southmead Hospital local research ethics committee. All analyses were carried out using STATA software version 8.0 (StataCorp, Texas, USA). Data were obtained from the Stork maternity information system used at this hospital, with the data from 2001 to 2004 used to derive baseline reference rates which were used in designing systems for monitoring deliveries in 2005. We included term (>37 weeks gestation), singleton, cephalic presenting live deliveries, and excluded all elective caesarean sections and home deliveries: the same inclusion and exclusion criteria used by Draycott et al[13].

Although the data analyses were all carried out retrospectively, a prospective scenario was created by serially inspecting the data taking into account the date and time of birth. Whenever a signal occurred, the process was stopped to allow for appropriate evaluation. In prospective monitoring, this would trigger an investigation into the cause of the signal, followed by appropriate corrective action. The next phase of monitoring would then follow.

### Reference rates (baseline standards)

The overall rate of low Apgar scores for 2001 to 2004 was found to be 0.44% (Table 1), the same as that published by Draycott et al[13]. An attempt to derive adjusted estimates of the risk of low Apgar from these data, using logistic regression found only "parity status" to be statistically significant. The final model obtained was however of a poor fit (R^2 ^= 0.009), and was not used. Stratified analyses were then carried out for the two groups, the nulliparous and the multiparous women. The baseline risks of low Apgar scores for these separate groups were 0.60% for nulliparous and 0.29% for multiparous women (Table 1). The CUSUM charts were then applied to the respective 2005 deliveries data separately.

**Table 1:** Estimates of the probability (risk) of low Apgar under the null and alternative hypotheses

	Risk of low Apgar under *H*_0_: (*OR *= 1)	Risk of low Apgar under *H*_*A*_: (*p given OR*_*A *_= 0.5)	Risk of low Apgar under *H*_*A*_: (*p given OR*_*A *_= 1.5)
Nulliparous	0.0060 (0.60%)	0.0030 (0.30%)	0.0089 (0.89%)
Multiparous	0.0029 (0.29%)	0.0015 (0.15%)	0.0044 (0.44%)
Overall	0.0044 (0.44%)	0.0022 (0.22%)	0.0066 (0.66%)

### CUSUM Chart settings

Two-sided Log-Likelihood CUSUM charts were used, with the upward chart designed to detect a 50% increase (*OR*_*A *_= 1.5) and the downward charts designed to detect halving (*OR*_*A *_= 0.5) of the odds of low Apgar scores. Determination of the unacceptable outcome rates, and hence the value of *OR*_*A *_for the Upward CUSUM as well as the limits took into account the results presented by Draycott et al, where the rates of low Apgar scores decreased from 0.86% to 0.44%[13]. This new overall rate of 0.44% (1 in 227) was regarded as the desired baseline rate which we wished to maintain, or indeed improve on. For the 2001 to 2004 data, we found the separate corresponding rates to be 0.29% (1 in 334) and 0.60% (1 in 167) for the multiparous and nulliparous women respectively. These, as well as the predicted rates under alternative hypotheses are illustrated in Table 1.

The CUSUM charts limits were placed at +1 and -1. A simulation approach was used to evaluate the in-control and out-of-control ARLs for these settings. The relevant ARLs are presented in Table 2, with corresponding values for limits at +1.5 and -1.5 also shown for comparison.

**Table 2:** Log Likelihood CUSUM chart performance – limits and corresponding ARLs

	*p*_0_	*p*_1_	*h*	IC-ARL_U_	IC-ARL_D_	Combined IC-ARL	OC-ARL_U_	OC-ARL_D_	Combined OC-ARL
Nulliparous	0.0060	0.0090	1.0	1730	1030	650	710	550	310
Multiparous	0.0029	0.0043	1.0	3500	2080	1300	1460	1140	640
Nulliparous	0.0060	0.0090	1.5	4420	2250	1490	1300	980	560
Multiparous	0.0029	0.0043	1.5	9390	5240	3360	2710	2005	1150

Where necessary, conversions between the odds and probability (risks) of low Apgar were made using the following formulae;

Odds low Apgar = *p/*(1 - *p*)

Probability of low Apgar under *H*_*A *_= *OR*_*A*_*p*_*t*_/(1 - *p*_*t *_+ *OR*_*A*_*p*_*t*_)

## Results

Out of a total of 18581 deliveries in 2001 to 2004, 15055 (81.02%) met the inclusion criteria and were used in the generation of baseline reference rates of low Apgar scores. There were 5010 deliveries in 2005, and 4028 (80.40%) of them were included and were thus monitored.

### Nulliparous women

**Figure 1:**
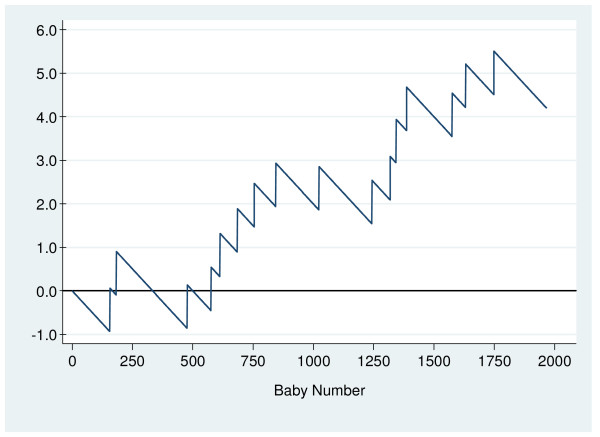
O – E chart for nulliparous deliveries.

**Figure 2:**
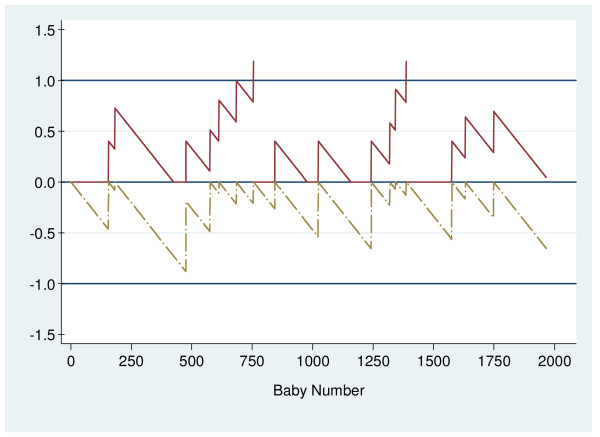
Log-likelihood ratio charts for nulliparous deliveries; Signals at Baby Numbers 755 and 1387.

The O – E CUSUM chart (Figure 1) shows that the rate of low Apgar scores for nulliparous women was generally higher in 2005 than in 2001 to 2004. Two upward CUSUM signals were observed at the 755^th ^and 1387^th ^deliveries (Figure 2). Table 3 shows a summary of results from the different phases of monitoring for nulliparous women. The rates of low Apgar scores during the two periods leading to signals were 0.93% and 0.95%, respectively. The overall low Apgar scores rate for nulliparous deliveries for the whole of 2005 was 0.81%.

**Table 3:** Nulliparous deliveries: Details of alarms and action taken

	Alarm details	Conclusion and Action
Phase 1: 1 – 755	Upward alarm at 755^th ^delivery Rate: 7 in 755 (0.93%)	Increase in rate of low Apgar scores Look for cause and take corrective action Continue monitoring (*p*_0 _= 0.0060, *h*^+ ^= 1)
Phase 2: 756 – 1387	Upward alarm at 1387^th ^delivery Rate: 6 in 632 (0.95%)	Increase in rate of low Apgar scores Look for cause and take corrective Continue monitoring (*p*_0 _= 0.0060, *h*^+ ^= 1)
Phase 3: 1388 – end	No alarms Rate: 3 in 579 (0.52%)	Continue monitoring (*p*_0 _= 0.0060, *h*^+ ^= 1)

### Multiparous women

**Figure 3:**
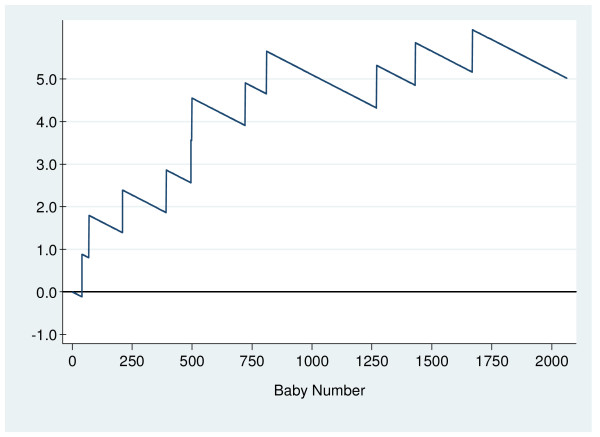
O – E chart for multiparous deliveries.

**Figure 4:**
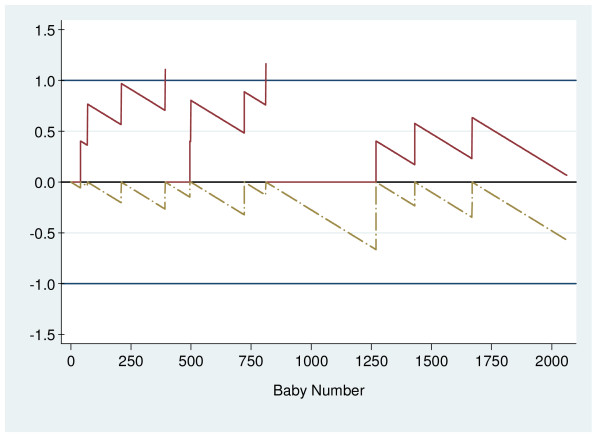
Log-likelihood ratio chart for multiparous deliveries; Signals at Baby Numbers 393 and 811.

The O – E CUSUM chart for multiparous women (Figure 3) shows that the rate of low Apgar scores was generally higher in 2005 than in 2001 to 2004. There were two signals at the 393^rd ^and the 811^th ^deliveries (Figure 4), with respective low Apgar score rates of 1.02% and 0.96% at each period. Table 4 summarises results from monitoring multiparous deliveries. The low Apgar scores rate for multiparous women for the whole of 2005 was 0.53%.

**Table 4:** Multiparous deliveries: Details of alarms and action taken

	Alarm details	Conclusion and Action
Phase 1 1 – 393	Upward alarm at 393^rd ^delivery Rate: 4 in 393 (1.02%)	Increase in rate of low Apgar scores Look for cause and take corrective action Continue monitoring (*p*_0 _= 0.0029, *h*^+ ^= 1)
Phase 2 394 – 811	Upward alarm at 811^th ^delivery Rate: 4 in 418 (0.96%)	Increase in rate of low Apgar scores Look for cause and take corrective action Continue monitoring (*p*_0 _= 0.0029, *h*^+ ^= 1)
Phase 2 811 – end	No alarm Rate: 3 in 1251 (0.24%)	Continue monitoring (*p*_0 _= 0.0029, *h*^+ ^= 1)

## Discussion

We have shown that a CUSUM based surveillance system can be used in monitoring healthcare outcomes, using routinely collected data. The O – E CUSUM chart provided a visual representation of the general trends in rates, while the Log-likelihood CUSUM chart provided ongoing formal statistical evaluations after each delivery, comparing the "current" rate with the baseline standard (reference) rate. Upward signals were observed, and the low Apgar score rates were confirmed to have risen significantly above expected rates. If such a system had been in prospective use at the time, detection of these periods could have been an opportunity for remedial action, potentially preventing some of the subsequent cases that were observed. Overall, there were 27 cases of low Apgar scores observed during the year, ten more than the number that would be expected from standard baseline rate of 0.44%.

The occurrence of a signal does not necessarily imply deterioration in performance. A signal indicates a change in the outcome rate, the cause of which can only be determined through subsequent investigations. All investigations should begin with checking the data for accuracy before taking any further steps, and for the results to be reliable the data need to be accurate. The quality of routinely collected data in the NHS has been found to vary from place to place. Data contained in one of the maternity information systems used in the UK has been shown to be of high quality [14,15], in direct contrast to data from other specialties [16]. Efforts at improving the quality of data should therefore be prioritised if the benefits of methods such as the CUSUM are to be realised.

The CUSUM technique has several methodological characteristics that should make it perform better than the commonly used healthcare quality control tools such as incident reporting and clinical audit, or indeed any other methods that report aggregate results. Unlike adverse incident reporting which has been shown to be incomplete and insensitive [17,18], the complete inspection of all relevant data ensures that no cases are missed. The retention of memory of recent results allows the CUSUM to detect even the small persistent shifts in rates which are otherwise easily missed. Unlike aggregate methods where poor runs can be compensated for and hidden by the existence of excellent results from elsewhere, the serial and continuous nature of data inspection allows the CUSUM to detect both good and bad runs separately as they occur, providing an opportunity for corrective action.

In common with all statistical tests, the CUSUM technique is associated with false positive and false negative states. A false positive is when the CUSUM gives an alarm when in actual fact performance is at an acceptable level and the rate of the poor outcomes has not changed. A false negative state exists when no alarms occur in the presence of suboptimal performance. The choice and placement of chart limits determines which of the two states is more likely to occur, and reducing the likelihood of one increases that of the other. This is connected to the determination of the ARL, with an ideal CUSUM design being one where the OC-ARL is short, and the IC-ARL is sufficiently long. In monitoring healthcare outcomes, a more sensitive system, i.e. one with shorter OC-ARL is to be preferred. In our situation, the low frequency of adverse outcomes as well as the small absolute differences between the desired and the undesirable rates places greater emphasis on greater sensitivity (hence lower limits). Higher limits could easily allow a return to the previous rates without this change being detected. In summary, for the nulliparous deliveries, there would be a signal after an average of 1730 deliveries, even if the rate of low Apgar scores had not increased (Table 2). For multiparous deliveries, the corresponding value is 3500. Corresponding ARL values for limits at +1.5 and -1.5 are also shown for comparison.

### Case-mix adjustment

Adjustment for case-mix is an important development to be added to the CUSUM methodology, and should ideally be used. Care is however required as risk-adjustment can be associated with a number of problems. Firstly, poorly fitting models may result from issues such as the absence of data on an important confounder. Secondly, the shelf life of even the best fitting models may be limited due to the dynamism of healthcare, where groups previously found to be at high risk of a certain outcome can quickly change to a different level of risk due to changes in practice.

In our study, a poorly fitting risk adjusted model was obtained and has not been used. Given the low event rate of our outcome, the large uncertainty in the model predictions could overshadow the changes in the rate of low Apgar scores. Adjustment through stratification of subjects into more homogeneous groups (nulliparous and multiparous) was considered an appropriate alternative option and was used here. Stratification, however, has its own disadvantages. The number of subjects in the groups being monitored is reduced and as a result, the power to detect small changes with any certainty, especially with a low event rate, is reduced. Increasing the number of groups that have to be simultaneously monitored results in increased risks of false signals due to problems of multiple testing[19]. In our case, a large number of deliveries were available and they were stratified to only two groups.

## Conclusion

A CUSUM based surveillance system can be used in continuous monitoring of clinical outcomes such as the low Apgar score, using the readily available "routinely collected data". This system could allow hospitals opportunities to improve standards of care for the patients in their care. Further research in a prospective setting is however required in order to fully evaluate these tools. Such evaluations should address the practicalities of using such systems as well as their clinical and cost effectiveness before wider uptake is advised.

## Competing interests

The author(s) declare that they have no competing interests.

## Authors' contributions

NS formulated the original concept and helped refine the hypothesis. TS refined the hypothesis, planned and designed the study, obtained and analysed the data, interpreted the results and wrote the initial draft. Both authors edited all drafts and read and approved the final manuscript. TS is the guarantor.

## Pre-publication history

The pre-publication history for this paper can be accessed here:


